# Factors Associated with Asthma Medication Adherence in Parents with Asthmatic Children: Theory of Planned Behaviour

**DOI:** 10.3390/healthcare13131613

**Published:** 2025-07-05

**Authors:** Ahmed M. Alshehri, Yasser S. Almogbel, Saud M. Alsahali, Yousif A. Alosaily, Ghada M. Almohaimeed, Lamis I. Alotayk, Abdulrahman A. Alqunaisy

**Affiliations:** 1 Clinical Pharmacy Department, College of Pharmacy, Prince Sattam Bin Abdulaziz University, Al-Kharj 16273, Saudi Arabia; ah.alshehri@psau.edu.sa; 2 Department of Pharmacy Practice, College of Pharmacy, Qassim University, Buraidah 51452, Saudi Arabia; s.alsahali@qu.edu.sa; 3 Maternity and Children Hospital, Qassim Health Cluster, Ministry of Health, Buraidah 52384, Saudi Arabia; yalosaily@moh.gov.sa; 4 Department of Pharmacology and Toxicology, College of Pharmacy, King Saud University, Riyadh 11451, Saudi Arabia; gha1996dah@gmail.com; 5 Department of Pharmacy, King Fahd Specialist Hospital, Buraydah 52719, Saudi Arabia; lamisibrahim.e1@gmail.com; 6 Community Pharmacist, Jamiah Alber, Almithnab 56646, Saudi Arabia; abdulrahman.qunaisy@gmail.com

**Keywords:** asthma, medication, adherence, parents, children, factors, theory of planned behaviour

## Abstract

Background/Objectives: Asthma is a prevalent chronic condition affecting approximately 300 million people globally. Despite advancements in treatment protocols, poor adherence to asthma medication remains a significant issue, often leading to severe complications, especially in children. This study aimed to identify factors influencing medication adherence among parents of children with asthma, using constructs from the theory of planned behaviour to better understand and improve adherence. Methods: This cross-sectional study employed a survey incorporating constructs from the theory of planned behaviour, demographic variables, and other adherence-related factors. Descriptive statistics and logistic regression analyses were applied to explore the relationship between these factors and adherence to asthma medications. Results: Out of 152 parents who visited the survey link, 150 were eligible. The average age was 35.58 ± 9.913 years; most participants were married (82%) and female (62.6%). Multivariate linear regression analysis of the parents’ factor showed parental attitude (β = 0.38, *p* < 0.001) and subjective norms (β = 0.34, *p* < 0.002) were significantly associated with parents’ intention to adhere to asthma medications. Conclusion: The study found that parental attitudes and subjective norms significantly impact the intention to adhere to asthma medication. Improving adherence is crucial for effective disease management, reducing healthcare costs, and enhancing the quality of life for children and their families. Interventions should focus on educating parents about the importance of adherence and engaging more family members to positively influence adherence through strengthened subjective norms.

## 1. Introduction

Asthma is a common, chronic disease. Over the last century, there has been a steep increase in the global prevalence of asthma [1]. Asthma impacts an estimated 300 million individuals worldwide and is associated with approximately 1000 daily fatalities [2]; the number of deaths in 2019 was 455,000 [3]. The majority of these deaths occurred in low- and middle-income countries, and most of these deaths were preventable [2]. Asthma is the most prevalent chronic condition in children globally [1] and remains a leading cause of non-communicable disease-related mortality, accounting for an estimated 12,900 child deaths worldwide in 2019 [4]. This underscores the urgent need for improved prevention and management strategies.

García-Marcos et al. conducted a study from 2015 to 2020, finding that asthma prevalence reached 11% in children aged 6–7 years and 9.1% in children aged 13–14 [5]. According to a study published in 2020, it was estimated that approximately 6.4 million children in the United States suffer from asthma [1]. Asthma exhibits a higher prevalence among males during childhood, whereas in adulthood, it is more frequently observed in females [6]. This disparity suggests a potential role of sex hormones during puberty in the aetiology of asthma subtypes [6].

In Saudi Arabia, asthma affects over two million individuals [7]. In a study conducted between 1986 and 2017, Alahmadi et al. reported that past studies have used the International Study of Asthma and Allergies in Childhood questionnaire to identify asthma prevalence rates ranging from 4–33.7% among children in Saudi Arabia [8]. The increase in asthma cases may be caused by rapid lifestyle changes associated with Saudi society’s modernization and exposure to environmental factors such as dust, indoor allergens, tobacco, and sand storms [9]. A sedentary lifestyle makes people spend more time indoors; as a result, exposure to allergens, such as mold, dust mites, and dust, may increase the chance of asthma episodes [10].

Despite advancements in asthma treatment in recent decades, improvements in patient education should be considered in managing asthma therapies [6]. In addition, the rising prevalence of asthma could be associated with an increase in asthma awareness among healthcare workers and the general population, allowing more individuals to be diagnosed [11]. Although there is no known cure for asthma, it can be managed. Successful long-term asthma management requires adherence to various health behaviors, including daily medication [12]. Healthcare providers can help children with uncontrolled asthma [13]. Despite various available treatments for asthma, many patients do not achieve the treatment goals due to non-adherence to their medication schedule, which leads to uncontrolled asthma episodes [14]. Consequently, this strains healthcare services, increases medical care costs, and decreases patients’ quality of life [14].

Special attention must be given to medication non-adherence in children and adolescents, as they encounter unique adherence challenges due to age. Young children rely on their parents for administering asthma medications, making adherence strongly dependent on parental motivation, attitudes, and the home environment [15].

Behavioral models such as the theory of planned behavior (TPB) can be used to predict the intention to do something (i.e., adherence). This model measures three predictors: attitude, subjective norms, and perceived behavioral control. First, attitude toward a behavior is a comprehensive evaluation of the behavior by determining whether the person favors doing so. Second, the subjective norm is a person’s estimate of the social pressure to perform a specific behavior. Finally, perceived behavioral control measures a person’s ability to perform a behavior [16,17].

Several factors affect medication adherence in children diagnosed with asthma. Some of these factors may be related to the children, and some to their parents [18]. In a study published in 2003, children’s age, sex, household size, and age at diagnosis were statistically associated with asthma medication adherence [18]. The study of 106 children with asthma and their parents showed that asthma medication adherence was lower in older children than in younger children [18]. A 2016 study conducted in New Zealand on 101 children presenting to a regional emergency department with asthma exacerbation reported that female children who had a smaller household size and were diagnosed with asthma at a younger age were strongly associated with superior asthma medication adherence [19].

Asthma medications, when taken correctly, reduce hospitalizations and emergency room visits. However, poor medication adherence eventually leads to uncontrolled asthma, severe asthma exacerbation, hospitalization, and increased morbidity and mortality [20].

Previous studies have reported that lack of knowledge about asthma medication among parents was associated with reduced medication adherence and led to poor control of asthma in children [21,22,23]. Since the primary caregivers for children with asthma are parents or legal guardians, parents need to understand the challenges that these children face with treatment and drug adherence.

A study conducted in Riyadh, Saudi Arabia, in 2015 on 292 parents of Saudi children with asthma reported that income level was associated with adherence [24]. Parents with lower incomes were more concerned about the adverse effects of inhaled corticosteroids and dependency on drugs, which represent a significant barrier to adherence to treatment and asthma care [25]. Although there are a few studies from Saudi Arabia about medication adherence among parents of asthmatic children, this is the first study to use the theory of planned behavior (TPB) model. Applying the TPB model would capture a wide range of psychological and social factors—including attitudes, subjective norms, and perceived behavioral control—that are essential for understanding what drives parental intention. This study aimed to predict the factors affecting medication adherence among parents of children with asthma in Saudi Arabia using the TPB.

## 2. Materials and Methods

### 2.1. Study Design and Data Source

This observational, cross-sectional study investigated the effects of attitude, subjective norms, perceived behavioral control of the TPB model, and other factors on parental adherence to asthma medications in children. Data were collected from February 2020 to May 2020 using a pre-tested online survey. The questionnaire was uploaded to an online survey system (Google Forms), and the link was advertised through social media platform “X” (formerly known as Twitter). This approach was chosen to ensure broad outreach and accessibility to parents of children diagnosed with asthma across different regions in Saudi Arabia. In order to maximize the sample size, and in addition to utilizing online recruitment, printed flyers with QR codes linked to the survey were distributed in two major hospitals in the Qassim region. Prior to data collection, ethical committee approval was obtained from the Central Institutional Review Board of the Ministry of Health, Riyadh (20-04E). Data were collected using a convenient sampling technique. Participants received 15 Saudi Riyals (SAR), equal to approximately $4, in prepaid telecom cards, or donated the same amount to a charity as an incentive.

When the participants clicked the link or scanned the QR code, they received an informed consent form regarding their participation. All eligible participants met the following criteria: (a) having asthmatic children aged 12 years or younger, and (b) willingness to participate in the survey. Once participants had agreed to participate, the website transferred them to the survey. After completing the survey, a delinked link was provided to transfer the participants to another website that was not linked to the survey due to privacy concerns. At the delinked link, participants entered their names and phone numbers to receive a gift or charity receipt as an incentive.

Based on an alpha level of 0.05, an effect size of 0.15, a power of 0.8, and 15 independent factors, a minimum sample size of 139 participants was calculated using the G*Power software(Version 3.1) [26].

### 2.2. Development of Questionnaire

First, the questionnaire was designed in English. The survey consisted of 37 questions divided into seven sections. In this study, the variables considered were the demographic characteristics of both parents and children, their attitudes, subjective norms, and perceived behavioral control. After developing the first draft, a questionnaire was administered to two experts for evaluation: a family physician consultant and a doctorate-holding individual in pharmacy practice. They were requested to provide their insights regarding the questionnaire, specifically assessing face validity and content validity, as well as identifying any questions that may be unclear or misleading. The survey was revised in accordance with the feedback received from the reviewers, and an evaluation was subsequently conducted. The final version of the questionnaire was translated from English to Arabic, followed by a subsequent back-translation [27], which was pretested on ten participants. To ensure understanding, clarity, suitability, and applicability, these factors were not included in the final data analysis.

The TPB model proposes three conceptually independent factors to measure the intention to commit to a health behavior, as the intention is considered the direct antecedent of behavior [16]. These factors are attitude (behavioral beliefs), subjective norm, and perceived behavioral control, which were the primary predictor variables evaluated in this study. The attitude question predicts whether the parent or caregiver favors giving their child asthma medication. Attitude was measured using bipolar adjectives, which were evaluated through four questions on a seven-point Likert scale (e.g., 1 = harmful to 7 = beneficial). Subjective norm questions measured the level of agreement with parents’ or caregivers’ social pressure to give asthma medications to their child, using four questions with a seven-point Likert scale, ranging from 1 (strongly disagree) to 7 (strongly agree). Perceived behavioral control questions measure parents’ or caregivers’ ability to adhere to the child’s medications. This was estimated using four questions, followed by seven Likert-scale choices. Parents’ confidence in adhering to their children’s asthma medications was assessed using self-efficacy and beliefs about the controllability of their behavior. The self-efficacy of parents was evaluated through a dual-item assessment, wherein parents were asked to report on two aspects: the perceived difficulty of administering the medication and their confidence in performing this task. The first item gauged their level of agreement, while the second item measured the level of difficulty encountered. Parents’ controllability efficacy was assessed by asking them to report on whether providing the medication was within their control and whether factors beyond their control influenced their adherence, using a seven-point Likert scale (1 = strongly disagree to 7 = strongly agree). All domains were systematically aggregated to produce a comprehensive total, with a higher cumulative score reflecting a more favorable outcome. We collected information on the (1) asthmatic child’s age (continuous), (2) sex (male or female), (3) presence of other chronic diseases (yes and no), (4) number of current asthma and other medication(s) (quantitative variable), (5) health insurance coverage, which was divided into three categories (private, government, and none), and (6) the number of children in the household. Age was collected as a continuous variable by asking participants to provide their child’s date of birth. Sex was categorized as male or female. We asked the children about their general health condition and whether they had any other chronic diseases. The number of children with asthma who were taking other medications was collected as a continuous variable.

We collected information on (1) age, (2) sex, (3) marital status, (4) employment status, (5) combined household income, (6) education level, and (7) health conditions. Age was recorded as a continuous variable by asking participants to provide their date of birth. Sex was categorized as male or female. We asked participants to indicate their marital status by choosing four response choices (married, single, divorced, or widowed). For inferential statistics, the marital status covariate was recorded as married or unmarried (single, divorced, or widowed). Income was recorded as a continuous variable. Employment status was categorized as full-time, part-time, or unemployed. Similar to the children’s demographic characteristics, we asked if they had any chronic diseases by providing checkboxes with the most common chronic diseases.

### 2.3. Statistical Analysis

Descriptive statistics and univariate analyses were used to identify factors affecting adherence. A multiple linear regression model was used to determine the factors affecting adherence. Data were coded, entered into Microsoft Excel 2016, and analyzed using Stata version 16. Statistical p-value was set at *p* < 0.05 was considered significant.

## 3. Results

### 3.1. Demographic Characteristics of Study Sample

Two hospitals agreed to participate in the study. A total of 152 parents visited the survey link, of whom 150 completed the survey. The mean participants’ age was 35.5 ± 9.9 (Table 1). The average age of the children was 8.6 ± 4.5 years. Although all of the participants did not report income, average income was determined to be SAR 12,311.3 ± 11,168.9.

Most participants were female (n = 92; 62.6%). Most parents were married (n = 123; 82%), had an educational level of college or higher (n = 96; 64%), and were employed full-time (n = 79; 52.7%). More than two-thirds of the participating parents had a chronic disease (n = 127, 84.7%) (Table 2).

Table 3 details participant demographics. In this study, more than two-thirds of the children were male (n = 117; 78%) and covered by government insurance (n = 104; 69.3%). Most children took 1–2 asthma medications, with the percentage decreasing as the number of medications increased. The majority of the children in this study did not have other chronic diseases (n = 122; 81.3%).

### 3.2. Parent’s Factor Associated with Intention to Adhere to Medications

Univariate analysis showed parental attitude (β = 0.59, *p* < 0.001), subjective norms (β = 0.60, *p* < 0.001), and perceived behavior (β = 0.51, *p* < 0.001) were significantly associated with parents’ intention to adhere to asthma medications. Further, working and student respondents (β = 2.128, *p* = 0.036) and marital status (β = 2.563, *p* < 0.047) were associated with a positive association with intention to give medications. However, univariate analysis of child-related factors showed no significant association (Table 4 and Table 5).

Table 6 shows the results of the multivariate analysis, with parents’ intention to adhere as a dependent variable and all key constructs from the univariate analysis that were statistically significant as independent variables. Parental attitude (β = 0.38, *p* < 0.001) and subjective norms (β = 0.34, *p* < 0.001) were significantly associated with parents’ intentions to adhere to asthma medications.

## 4. Discussion

Effective asthma management involves a thorough assessment of the patient’s situation and appropriate medication prescribed by a physician, along with careful adherence to the treatment plan by the patient. Consequently, parental adherence to prescribed asthma medications for their children plays a pivotal role in the effective management of asthma in children. Our study predicted the effect of parents’ attitudes, perceived behavior, subjective norms, and parents’ and children’s demographics on their intention to adhere to children’s asthma medications. We found that parental attitudes and subjective norms affected the intention to adhere to medication for asthma. This is the first study conducted in Saudi Arabia to explore parents’ attitudes toward their children’s asthma medication using TPB.

The Saudi fertility rate has gradually shaped household size and family structure. Increasing numbers of unmarried, divorced, and working women have also changed Saudi Arabian homes [28]. Marriage, fertility, and livelihood changes are transforming households worldwide [29]. This was clear in this study based on parents’ age and the number of children in the household. The average age was 35.5 years, which might have been affected by the aforementioned factors, in addition to the fact that an asthmatic child might not be the first child between siblings. Another reason may be that the survey was completed using QR codes, which may not be widely used by older adults, who might have more children. Regarding the number of children, most families reported one child. The demographic composition of households underwent notable changes in 2016, characterized by a decline in the number of children (<5- and 5–14-year-old) and adolescents, offset by an increase in the adult and elderly segments [28]. Another reason may be that the survey was filled out using QR codes and advanced technologies, which are not used by older adults who might have more children. Additionally, most participants had college education, which may be due to how this survey was collected or because people with more education might care more about their children. The reported monthly income for parents (SAR 12,311.3) was more than the national average (SAR 10,238) ($2730) [30]. This variation may be because the sample included older adults who had worked for years. More than half of the participants’ children were boys. This is in agreement with a study conducted at the national level that included 11,348 adolescent participants, which found that most asthmatic patients were males [31]. This finding may be due to the fact that the number of boys is greater than that of girls in Saudi Arabia, according to the statistics released by the General Authority for Statistics in 2020 [32]. Over half of the participants were covered by government healthcare. Healthcare is provided to Saudi citizens at no cost through the Ministry of Health (MOH) and other governmental healthcare systems [33].

Patient adherence is of interest to many researchers today, and several studies have successfully demonstrated a strong relationship between patient beliefs about medicines and medication adherence. While some studies have assessed patients’ personal beliefs and their impact on adherence [34,35,36], others have specifically examined how parental beliefs influence adherence to medications in children [37,38]. For example, one study found that parents with greater concerns about asthma medications were significantly more likely to report poor adherence in their children [37]. Conversely, other research has shown that poor adherence may persist in children despite high levels of concordance between healthcare providers and parents regarding illness perceptions and medication beliefs, even in the absence of socioeconomic barriers [38]. These findings suggest that while parental medication beliefs are important, additional factors—such as routines, family dynamics, and behavioral barriers—may also play a critical role in shaping adherence behaviors in children asthma management.

Our findings are consistent with those of previous studies. We found a significant association between parental attitude and intention to adhere; therefore, parents with a negative attitude toward adherence are less likely to adhere to asthma medication regimens [39]. We found that social pressure on parents has a significant and positive relationship with their intention to adhere. Most parents feel pressured by people who were important to them to adhere to their children’s medications for asthma. This finding is in line with previous studies by Gonzalez et al. and Pan et al., who found that medication adherence improves as social pressure increases [40,41]. A study conducted among patients with asthma aged 12 to 20 years from the USA revealed a significant connection between non-adherence to medication and a decrease in perceived social support [42]. Moreover, a study of 75 adult patients in the USA taking antiretroviral medication found that higher social support was associated with higher adherence to antiretroviral medications [43].

Asthma ranks 19th in disability-adjusted life years (DALYs) and 26th in deaths in Saudi Arabia, affecting two million Saudis, with most of them having uncontrolled conditions that adversely affect their quality of life [44,45]. Hence, parents’ commitment to adherence is paramount in controlling the disease. This will ultimately lead to a decrease in the impact of the disease on quality of life and a reduction in asthma-related mortality rates.

Our study had a few limitations. Most notable, the observed associations do not imply causality, mainly because the data were collected at a single point. The generalizability of these findings may be limited due to the use of convenience sampling, which can compromise a sample’s representativeness. This limitation increases the risk of sampling errors and misinterpretation of the data. Convenience sampling may also lead to undercoverage bias, as it typically includes only those who are easily accessible, thereby excluding other segments of the population. Additionally, it may introduce self-selection bias, since individuals who voluntarily choose to participate often differ systematically from those who do not. It is imperative to conduct further investigations across the diverse regions of Saudi Arabia using more rigorous sampling methods or applying strategies to reduce bias. Moreover, it is critical to acknowledge that our study was conducted with a relatively modest sample size; greater statistical power could be achieved in a larger cohort. Nonetheless, it is pertinent to highlight that the pursued sample size was predicted by appropriate calculations. Finally, the survey was distributed using QR codes and online platforms, which may have limited participation from older parents who are less familiar with such technologies.

## 5. Conclusions

This study investigated the impact of parental attitudes and subjective norms on their intention to adhere their children to asthma medication schedules. A significant association was found between parents’ intention to adhere their children to asthma medications and their attitudes and subjective norms among parents of asthmatic children in Saudi Arabia. The significance of enhancing adherence lies in the capacity to effectively manage the disease, curtail costs, and improve patients’ quality of life by mitigating asthma symptoms that impede their daily activities. Parents’ low attitudes and subjective norms toward adherence emphasize the need for an educational clinic to expand their understanding of the importance of adherence, which will improve their attitudes. To improve adherence, it is advisable to involve a greater number of family members, particularly parents and other influential individuals, in the decision-making process.

## Figures and Tables

**Table 1:** Continuous Baseline Characteristics of Participants’ Parents (n = 150).

Parent Characteristics	Mean (±SD)
Parents’ age	35.6 (9.9)
Income in SAR (1 SAR = 0.266 US dollar)	12,311.3 (11,168.9)
Children’s age	8.6 (4.5)

**Table 2:** Categorical Baseline Characteristics of Participants’ Parents (n = 150).

Parent Characteristics	n (%)
Sex	
Male	55 (37.4)
Female	92 (62.6)
Marital Status	
Married	123 (82)
Not married	27 (18)
Employment status	
Full time	79 (52.7)
Part-time	11 (7.3)
Not employed	60 (40)
Education level	
Graduated or elementary school level and below	2 (1.3)
Graduated from intermediate school	5 (3.3)
Graduated secondary school	25 (16.7)
Graduated with diploma	22 (14. 7)
Graduated from college	77 (51.3)
Graduated at Master of PhD level	19 (12.7)
Presence of chronic disease	
Yes	127 (84.7)
No	23 (15.3)
Relationship to the child	
Mother	67 (51.1)
Father	49 (37.4)
Other	15 (11.5)

n = number of participants.

**Table 3:** Categorical Baseline Characteristics of Children with Asthma (n = 150).

Child Characteristics	n (%)
Sex	
Male	117 (78)
Female	33 (22)
Other Chronic diseases	
Yes	28 (18.7)
No	122 (81.3)
Number of child medications	
One	53 (35.3)
Two	52 (34.7)
Three	28 (18.7)
Four or more	17 (11.3)
Number of children	
One	79 (52.7)
Two	11 (7.3)
Three	6 (4)
Four or more	54 (36)
Health insurance coverage	
Private	27 (18)
Governmental	104 (69.3)
None	38 (25.3)

n = number of participants.

**Table 4:** Univariate Linear Regression Analysis of Parental Factors Associated with Medication Adherence Intentions.

Independent Variable	β	95% CI	*p*-Value
Attitude	0.597	0.459–0.734	<0.001 *
Subjective norm	0.605	0.450–0.761	<0.001 *
Perceived behavior	0.514	0.295–0.734	<0.001 *
Parents’ age	0.12	0.017–0.223	0.023
Caregiver	1.164	−3.918–6.244	0.652
Mother is the Caregiver	0.233	−1.748–2.213	0.817
Other caregivers	Ref.		
Education level:			
College diploma and more	1.013	−1.386–3.411	0.653
Less than a college diploma	Ref.		
Working or student			
Yes	2.128	0.135–4.120	0.036 *
No	Ref.		
Marital status			
Married	2.563	0.034–5.092	0.047 *
Non-married	Ref.		
Family members working in the medical field			
Yes	0.626	−1.341–2.592	0.531
No	Ref.		
Parent’s chronic disease:			
Yes	1.072	−0.990–3.134	0.306
No	Ref.		

Note: * *p* < 0.05. Abbreviations: β, beta coefficient; 95%CI, 95% confidence interval; Ref., reference.

**Table 5:** Univariate Linear Regression Analysis of Child Factors Associated with Parents’ Medications Adherence Intentions.

Independent Variable	β	95% CI	*p*-Value
Child age	0.075	−0.146–0.295	0.503
Male child	2.099	0.253–4.452	0.08
Sever	0.022	0.043–0.087	0.507
Number of years of asthma diagnosis	0.626	−1.345–2.593	0.531
Number of children in a family	−0.051	−0.543–0.441	0.837 0.742
Number of medication			
One preventive medication	−0.845	−2.901–1.210	0.418
More than medication	Ref.		
Payment of health service			
Free	−0.528	−2.662–1.606	0.625
Paid	Ref.		
Receive health care services by:			
Free clinics (e.g., community) or government clinics)	−0.371	−2.365–1.623	0.714
Private hospitals and clinics	Ref.		
A pediatric care physician whom you regularly see for your asthmatic child	0.226	−1.896–2.349	0.833
A specific pharmacist who regularly answers your child’s medication-related questions	0.637	−1.644–2.919	0.582
Child’s chronic disease:			
Yes	1.66	−0.889–4.209	0.2
No	Ref.		

Note: Abbreviations: β, beta coefficient; 95%CI, 95% confidence interval; Ref., reference.

**Table 6:** Multivariate Linear Regression of Factors Associated with Parental Medication Adherence Intentions.

Intention	β	95% CI	*p*-Value
Attitude	0.387	0.165–0.608	0.001 *
Subjective norm	0.35	0.135–0.565	0.002 *
Perceived behavior	−0.047	−0.295–0.201	0.709
Caregiver age	0.055	−0.045–0.154	0.277
Married Caregiver			
Yes	1.067	−1.393–3.526	0.392
No	Ref.		
Working caregiver or student			
Yes	1.105	−0.610–2.820	0.205
No	Ref.		
Child’s sex			
Male	1.79	−0.324–3.903	0.096
Female	Ref.		

Note: * *p* < 0.05. Abbreviations: β, beta coefficient; 95%CI, 95% confidence interval; Ref., reference.

## Data Availability

The data that support the findings of this study are available from the corresponding author.
